# Male participation in prevention programmes of mother to child transmission of HIV: a protocol for a systematic review to identify barriers, facilitators and reported interventions

**DOI:** 10.1186/2046-4053-1-13

**Published:** 2012-02-16

**Authors:** Frederick LI Morfaw, Lehana Thabane, Lawrence CE Mbuagbaw, Philip N Nana

**Affiliations:** 1 Department of Obstetrics and Gynaecology, Faculty of Medicines and Biomedical Sciences, University of Yaoundé 1, PO Box 1364, Yaoundé, Cameroon; 2 Department of Clinical Epidemiology and Biostatistics, McMaster University, Hamilton, ON, Canada; 3 Biostatistics Unit, Father Sean O'Sullivan Research Centre, St Joseph's Healthcare - Hamilton, Hamilton, ON, Canada; 4 Centre for the Development of Best Practices in Health (CDBPH), Yaoundé Central Hospital, Henri, Dunant Avenue, Messa, PO Box 87, Yaoundé, Cameroon

**Keywords:** Male participation, PMTCT, HIV, systematic review protocol

## Abstract

**Background:**

Infection with the HIV and AIDS are leading causes of morbidity and mortality among women and children worldwide. Prevention of mother-to-child transmission of HIV (PMTCT) programs were developed to protect women and their babies from having HIV infection. However, knowledge on how male participation has been applied to these programs is limited. We present a research protocol for a review which seeks to determine the effects of male participation on female uptake of PMTCT programs, and assess how this male participation has been investigated and documented worldwide.

**Methods:**

This is a systematic review of published literature. We will attempt to identify all studies relevant to the subject written in the English language from January 1998 to June 2011. Electronic searches of the PubMED, EMBASE, CINAHL, and LILACS databases will be conducted using the relevant medical subject headings. Reference lists of identified studies and previous reviews will be manually checked for articles of interest. We shall also contact authors on the field for any relevant material. Two authors (FM and LM) will independently screen potential articles for eligibility using well-defined inclusion and exclusion criteria. They will independently assess the methodological quality of each included paper using the Jadad scale for randomized controlled trials, and the Newcastle-Ottawa scale for observational studies. Then they will independently extract data from the studies using a pre-established data extraction form. The primary outcome data will be female uptake of PMTCT services following a male/couple intervention, while secondary outcome measures will include indicators and barriers of male participation in PMTCT activities among others. During the data extraction process, discrepancies between the two authors will be sorted out by discussion or consultation with a third party (LT). The analysis and reporting of the review will be according to the PRISMA and MOOSE guidelines. Any identified clinical or statistical heterogeneity will be explored. Where possible, a random-effects meta-analysis will be performed to obtain aggregate estimates. We will also assess publication bias using funnel plots. Analysis of other outcomes will be descriptive.

## Background

Infection with the HIV and AIDS continue to be leading causes of morbidity and mortality among women and children worldwide [1]. According to 2008 estimates, over 430,000 children were newly infected with HIV, and over 90% of these were through mother-to-child transmission (MTCT) of HIV [2]. MTCT of HIV can occur during pregnancy, labour, delivery, and during breastfeeding. In the absence of any intervention there is a 20% to 45% transmission rate from mother to child, but this risk can drop to 2% in non-breastfeeding populations, and 5% in breastfeeding populations in which specific interventions are carried out [2]. These interventions include: providing lifelong antiretroviral treatment (ART) for all pregnant women with CD4 ≤350 cells/mm^3 ^or advanced clinical disease; providing combination antiretroviral (ARV) prophylaxis with either Zidovudine (AZT) or triple ARV prophylaxis beginning in the second trimester and linked to postpartum prophylaxis; providing prophylaxis to either the mother or the infant during breastfeeding in settings where breastfeeding is the preferred feeding option [2].

The Global Fund Board recognizes prevention of HIV infection among women, prevention of death among HIV positive women, and preventing babies from being infected with HIV, as crucial priorities [1]. Prevention of mother-to-child transmission (PMTCT) of HIV programs were developed since 1998 to protect women and their babies from infection. At present the World Health Organization (WHO) promotes a comprehensive four-pronged approach for PMTCT. This includes: preventing HIV infection among women of childbearing age; preventing unintended pregnancies among women living with HIV; preventing HIV transmission from a woman living with HIV to her infant; and providing appropriate treatment, care, and support to mothers living with HIV, their children, and their families [2]. These programs are more focused on women and generally omit men. The following pertinent questions therefore stand out: What is the effect of male participation in preventing MTCT of HIV among pregnant women, and how has this participation been documented worldwide?

Knowledge on how male participation has been applied to national PMTCT programs worldwide is limited. The male partner plays an important role in women's reproductive health and improvement of PMTCT outcomes. This role of the male partner in women's risk of acquiring HIV, in the uptake of voluntary counseling and testing (VCT) of HIV and PMTCT programs has been described in many studies [3-6]. Other observational studies indicate that educating men about the importance of family healthcare improves health-seeking behavior for antenatal care and child immunization [7,8] and also enhances communication and support of the female partner [9,10]. This evidence suggests that male partner involvement is crucial. The importance of male partner participation is also recognized by the WHO in their 2010 PMTCT Strategic Vision document where it is stated that "male partners play an equally important role in the scale-up of PMTCT services" [2].

In this era of the HIV/AIDS epidemic, more attention is directed towards incorporating men into reproductive health education interventions [11]. Involving men in PMTCT services could increase the uptake of couple counseling and disclosure of HIV status. This would open doors for the provision of services to HIV-negative couples and discordant couples, as well as preventive care and treatment for HIV-positive couples and their families [12]. Male involvement could enhance partner support for follow-up care for HIV-positive pregnant women and HIV-exposed infants, including ARV adherence, improved adherence to infant feeding methods, and early management of HIV-exposed infants [12]. It could also eliminate harmful consequences faced by women who seek PMTCT services such as stigmatization and gender-based violence. Moreover, male involvement in PMTCT services could address the healthcare needs and responsibilities of men, providing them with positive male norms, and linking them to other healthcare services [12].

This review seeks to determine the effect of male participation on female uptake of PMTCT services, and assess how male participation in PMTCT has been investigated, described, promoted, and documented worldwide. It will summarize the evidence for and against male participation in PMTCT programs and review the interventions which have been investigated in enhancing male participation in PMTCT activities. It would provide a solid evidence base for advocating increased male participation in PMTCT activities. It would also help formulate recommendations on how to increase male participation in PMTCT activities. From the evidence in the review, we plan to formulate a standardized Scale for the Assessment of Male Participation in PMTCT programs (SAMP-PMTCT). If validated, this scale would serve as a useful tool in assessing the level of male participation in PMTCT activities.

### Objectives

The primary objective of this systematic review is to determine the effect of male participation on female uptake of PMTCT services.

The secondary objectives are: to determine the percentage of men who accept voluntary counseling and testing for HIV, provide moral and financial support to their spouses to adhere to antenatal care (ANC) and PMTCT guidelines; to identify indicators of male participation in PMTCT activities (supportive and non-supportive); to evaluate men's knowledge and approval of PMTCT interventions; to evaluate determinants of male involvement in PMTCT and antenatal care; to evaluate interventions used to enhance male participation in PMTCT; to identify barriers to male participation in PMTCT programs; and to determine potential interventions for improving male involvement in ANC and PMTCT activities.

## Methods

### Criteria for considering studies for this review

#### Types of studies

Randomized controlled trials and observational studies will be selected for the review.

#### Study settings

Included studies must have been conducted in a context of ANC and/or PMTCT of HIV. We will use literature from any geographic or socioeconomic setting.

#### Types of participants

Participants shall include men, women, or focused groups of the community. Irrespective of the type of participants, we shall include studies that report outcomes that could possibly (theoretically) affect female uptake of PMTCT services, regardless of whether uptake data are reported.

#### Outcome measures

The primary outcome of this review will be female uptake of PMTCT services following a male/couple intervention.

Secondary outcomes would include: the proportion of men who accept voluntary counseling and testing for HIV, provide moral and financial support to their spouses to adhere to antenatal care and PMTCT guidelines; indicators of male participation in PMTCT activities; men's knowledge of PMTCT; men's knowledge of MTCT of HIV; proportion of men who approve PMTCT; determinants of male involvement in PMTCT programs and ANC; percentage of women receiving VCT for HIV and obtaining their results; percentage of HIV-positive pregnant women accepting ARVs; couple uptake of VCT; number of men coming for services as a result of provider invitation; barriers to male partner participation in ANC and PMTCT activities: we will collate all the barriers to male partner participation as reported by the authors; suggestions for improving male involvement in ANC and PMTCT activities.

### Search strategy for identification of studies

We shall attempt to identify all studies relevant to the review, written in English from the beginning of the year 1998 (year of introduction of PMTCT programs) to June 2011.

#### Electronic searches

We will search the PubMED, EMBASE, CINAHL, and LILACS databases for relevant articles written in English from January 1998 to June 2011. We will use the subject term services of the different databases. The following search terms and their MeSH (medical subject heading) equivalents will be used: HIV, male, spouse, partner, men, couple, pregnancy, gestation, participation, involvement, engagement, antenatal, barriers, facilitators, disclose, declare, testing, PMTCT, voluntary counseling and testing (VCT), vertical transmission, domestic violence. These terms will be used in varying combinations. The term HIV will be present in every search.

#### Reference lists

We will manually check the reference list of all the studies identified by the above search strategy. Relevant studies shall be observed, and where appropriate, included in the review.

We shall also check the reference list of any previous reviews on male participation and PMTCT for any relevant articles which could be included in the review.

#### Grey literature

We will attempt to contact authors, experts in the field, and research organizations for any relevant material.

### Data collection and analysis

#### Screening

Two authors (FM and LM), working independently, will screen all citations and abstracts identified by the search strategy to identify eligible papers. The full text of eligible articles will be obtained. These will be assessed using a pre-designed eligibility form based on the inclusion criteria. Eligible studies will be included in the review. Disagreements will be resolved by discussion or by seeking the opinion of a third party (LT).

Authors will be contacted in order to clarify missing aspects or unclear information.

Ineligible studies will be excluded from the review and the reason for exclusion stated in a table.

#### Data extraction

The two authors (FM and LM) will independently extract data from the studies using a pre-established data extraction form (see sample example in Additional file 1). Information obtained will include the site of the study, the purpose of the study, the study design, the study settings, participant characteristics, interventions if any, outcomes, forms of male participation, and so on. Discrepancies in the extracted data will be discussed by the two authors for consensus; if not, this will be reviewed by a third party (LT). In case of unclear or incomplete data, we will contact the authors for further details.

#### Assessment of methodological quality

Two authors (FM and LM) will independently assess the methodological quality of included studies using the Jadad scale [13] for randomized controlled trials, and the Newcastle-Ottawa scale [14] for observational studies. In case of doubt or missing details, authors will be contacted for clarification. Discrepancies will be discussed by the two authors for a consensus. If an agreement is not reached a third party (LT) will be consulted.

#### Measures of intervention effect

We shall use the Review Manager Version 5.1 software for analysis, and results will be presented using 95% confidence intervals. The odds ratio and the relative risk will be used to measure intervention effects for dichotonomous data. In case of continuous outcomes, the mean difference will be calculated when outcome measures in all studies are made on the same scale. However, if outcomes are measured in different scales, the standardized mean difference will be assessed.

#### Data synthesis, assessment/investigation of heterogeneity

We will assess the participants, methods, and intervention effects of included studies for heterogeneity. This will enable us determine whether results can be pulled across studies. Any identified clinical or statistical heterogeneity will be explored visually or using the chi square test. Possible sources of heterogeneity include geographical location (developed versus developing country), age groups, marital status, study design. In case of heterogeneity, subgroup analyses according to these different categories will be conducted. If included studies are similar enough to combine, a fixed-effect meta-analysis will be conducted. Otherwise, a random-effects meta-analysis will be performed to incorporate heterogeneity among studies and obtain aggregate estimates.

#### Sensitivity analyses

A sensitivity analysis will be performed to determine any bias introduced by the eligibility criteria, the data analyzed, the analysis method, and other relevant issues identified during the review process.

#### Presenting and reporting of results

The analysis and reporting of the review will be done according to the PRISMA [15] and MOOSE [16] guidelines. We will use a flow diagram to summarize the selection process of the studies. Agreement between data abstractors will be assessed using the kappa statistic [17].

We will also assess publication bias using funnel plots. A "summary of findings" table will be used to present the most important outcomes. Reporting of other outcomes will be descriptive.

## Discussion

### Expected significance of the study

From the evidence in the review, we hope to formulate in a subsequent project, a standardized Scale for the Assessment of Male Participation in PMTCT programs (SAMP-PMTCT). This scale would be objective and generalizable in a wide range of settings. During this subsequent project the validity and reliability of this scale will be tested and at the same time, it will be used to evaluate the level of male participation in PMTCT programs in our setting.

Moreover, the data derived from the review could inform the design of a randomized clinical trial on interventions aimed at improving partner attendance in PMTCT activities. We foresee an intervention arm which may include invitations to participate, financial incentives, home visits, and other motivational factors identified from the review. The control arm will consist of women who take part in PMTCT activities alone. Our outcomes would include acceptance of HIV screening, transmission rates to children, obstetric outcomes, and patient satisfaction. This would provide a solid evidence base for policy formulation on the integration of men in PMTCT activities. Once these policies are implemented, they could further help in preventing MTCT of HIV worldwide, and would be a major contributing step in the fight against HIV.

## Abbreviations

ANC: antenatal care; ART: antiretroviral therapy; ARV: antiretroviral; AZT: Zidovudine; CINAHL: Cumulative Index of Nursing and Allied Literature; EMBASE: Excerpta Medica DataBase; LILACS: Latin American and Caribbean Health Sciences Literature; MOOSE: Meta-analyses of Observational Studies in Epidemiology; MTCT: mother-to-child transmission; PMTCT: prevention of mother-to-child transmission; PRISMA: Preferred Reporting Items for Systematic reviews and Meta-Analyses; WHO: World Health Organization; VCT: voluntary counseling and testing.

## Competing interests

The authors declare that they have no competing interests.

## Authors' contributions

FM, LT, and LM jointly conceived the paper. FM made the first draft. FM, LT, LM, and NP reviewed several versions of the manuscript. All authors read and approved the final manuscript.

## Supplementary Material

Additional file 1 **General description of what to include in the data extraction form**. This form broadly describes the information which we hope to extract using the data extraction. It includes general information about the included studies, an assessment of the inclusion criteria, and brings out the interventions (if any) used in the studies. This form also has a section showing what was measured at the end of each study, the summary statistics that were used, and the result summaries in each case. Click here for file
