# Supplementary material for: Male participation in prevention programmes of mother to child transmission of HIV: a protocol for a systematic review to identify barriers, facilitators and reported interventions
Source: Syst Rev. 2012 Feb 16;1:13. doi: 10.1186/2046-4053-1-13 (PMC3351738; doi:10.1186/2046-4053-1-13)
Supplement: Additional file 1 — General description of what to include in the data extraction form. This form broadly describes the information which we hope to extract using the data extraction. It includes general information about the included studies, an assessment of the inclusion criteria, and brings out the interventions (if any) used in the studies. This form also has a section showing what was measured at the end of each study, the summary statistics that were used, and the result summaries in each case. [file 2046-4053-1-13-S1.DOC]

**ADDITIONAL FILE 1**

**GENERAL DESCRIPTION OF WHAT TO INCLUDE IN THE DATA EXTRACTION FORM**

**1 GENERAL INFORMATION**

- Date of extraction (yyyy/mm/dd):
- Identification features of the study
  - Author:
  - Article title
  - Source, Year/volume/pages/country of origin
  - Institution, affiliation or contact address
- Identification of the reviewer (FM, LM):
- Notes:

**2 INCLUSION/EXCLUSION CRITERIA**

- Article in English language: **YES/NO**
- Publication year (1998-2011): **YES/NO**
- Appropriate study design (Randomized controlled trial, Observational study): **YES/NO**
- Appropriate study settings (antenatal care, PMTCT): **YES/NO**
- Participants (Adult males and females above 15 years old, focused groups of the community): **YES/NO**
- Appropriate outcome measures: female uptake of PMTCT interventions (VCT, ARV prophylaxis, non breastfeeding), male VCT uptake, men's knowledge on PMTCT, men's support/approval of PMTCT, indicators of male PMTCT participation, number of men attending PMTCT services, male barriers to PMTCT interventions, interventions to enhance male PMTCT participation, incentives to increase male PMTCT participation,: **YES/NO**

**3 SPECIFIC INFORMATION**

- Re-verification of study eligibility: (correct population, design, outcomes): **YES/NO**
- Population characteristics and care settings
  - Target population (Men women, pregnant women, community):
  - Number of participants:
  - Recruitment procedures used ( participation rates if available):
  - Participants characteristics at the start of the study (sex, age, class, geographical region, other information):

**4 INTERVENTIONS**

- Focus of intervention (Males, couples, females, groups)
- Intervention site (hospital, community, other)
- Delivery mode of intervention (lectures, group discussions, individual counseling)

**5 OUTCOME MEASURES (what was measure at the end of the study):**

**a) Quantitative data**

- Number of pregnant women who receive HIV testing.
- Number of sero-discordant couples.
- Number of HIV positive pregnant women accepting ARV prophylaxis.
- Female uptake of PMTCT interventions (VCT, ARV prophylaxis, non-breastfeeding).
- Male VCT uptake (percentage).
- Number of men attending ANC/PMTCT services.
- Men's support/approval of PMTCT, (percentage).
- Number of men coming for services as a result of provider invitation.

b) **Qualitative data**

- Men's knowledge on PMTCT,
- Indicators of male PMTCT participation,
- Male barriers to PMTCT participation:
  - - *Individual barriers:*
    - *Family barriers:*
    - *Community barriers:*
    - *System barriers:*
- Facilitators of PMTCT participation
  - - *Individual facilitators:*
    - *Family facilitators:*
    - *Community facilitators:*
    - *System facilitators:*
- Interventions to enhance male PMTCT participation,
- Incentives to increase male PMTCT participation,

**6 ANALYSIS**

- Statistical technique used
- Does technique adjust for confounding? YES/NO
- Unit of analysis

**7 RESULTS**

- Quantitative results: e.g. estimates of a size effect (odds ratios, relative risks, p-values and 95% confidence interval, percentage change, mean difference, standard mean difference).
- Qualitative results: narrative of effects
